# Alternations of Blood Pressure Following Surgical or Drug Therapy for Prolactinomas

**DOI:** 10.3390/cancers16040726

**Published:** 2024-02-09

**Authors:** Yijun Cheng, Dapeng Wang, Hao Tang, Debing Tong, Weiguo Zhao, Shaojian Lin, Hong Yao, Wenwen Lv, Xun Zhang, Li Xue, Hanbing Shang, Zhe Bao Wu

**Affiliations:** 1 Department of Neurosurgery, Center of Pituitary Tumor, Ruijin Hospital, Shanghai Jiao Tong University School of Medicine, Shanghai 200025, China; cyj12574@rjh.com.cn (Y.C.); wdp2023@sjtu.edu.cn (D.W.);; 2 Department of Cardiology, The Third Affiliated Hospital of Soochow University, Changzhou 213003, China; 3 Clinical Research Center, School of Medicine, Shanghai Jiao Tong University, Shanghai 200025, China; 4 Neuroendocrine Research Laboratory, Massachusetts General Hospital and Harvard Medical School, Boston, MA 02114, USA; 5 Department of Neurosurgery, Ruijin-Hainan Hospital, Shanghai Jiao Tong University School of Medicine, Haikou 570312, China

**Keywords:** prolactinoma, hypertension, blood pressure, surgery, cabergoline, prolactin

## Abstract

**Simple Summary:**

Prolactinoma is the most common subtype and composes about 40–66% of all pituitary neuroendocrine tumors (PitNETs). Studies suggested that some other subtypes of PitNETs, such as Acromegaly and Cushing disease, could result in hypertension. However, this relationship remains unclear in prolactinoma. Our study revealed that in situ rat and xenograft nude-mice prolactinoma induced a significant BP increase, which was attenuated by cabergoline (CAB) treatment. In clinic, surgery decreased BP in prolactinoma patients both with or without hypertension. This BP-lowering effect was significantly associated with several variables, including age, sex, disease duration, tumor size, invasion, resistance to dopamine agonists (DAs), recurrence, and preoperative PRL levels.

**Abstract:**

Several subtypes of pituitary neuroendocrine tumors (PitNETs), such as acromegaly and Cushing’s disease, can result in hypertension. However, whether prolactinoma is associated with this complication remains unknown. Moreover, the effect of treatment with surgery or drugs on blood pressure (BP) is unknown. Herein, a retrospective study reviewed 162 patients with prolactinoma who underwent transsphenoidal surgery between January 2005 and December 2022. BP measurements were performed 1 day before and 5 days after surgery. Accordingly, patients’ medical characteristics were recorded. In addition, in situ rat and xenograft nude-mice prolactinoma models have been used to mimic prolactinoma. In vivo BP and serum prolactin (PRL) levels were measured after cabergoline (CAB) administration in both rats and mice. Our data suggest that surgery can effectively decrease BP in prolactinoma patients with or without hypertension. The BP-lowering effect was significantly associated with several variables, including age, sex, disease duration, tumor size, invasion, dopamine agonists (DAs)-resistance, recurrence, and preoperative PRL levels. Moreover, in situ and xenograft prolactinomas induced BP elevation, which was alleviated by CAB treatment without and with a statistical difference in rats and mice, respectively. Thus, surgery or CAB can decrease BP in prolactinoma, indicating that pre- and postoperative BP management becomes essential.

## 1. Introduction

Prolactinoma is the most common subtype of pituitary neuroendocrine tumors (PitNETs) and composes approximately 40–66% of all PitNETs, which also includes Acromegaly, Cushing disease, nonfunctioning pituitary adenomas, etc. [1,2]. It has been reported that this disorder affects as many as 6–10 people per 100,000 per year [3]. Although prolactinoma is a benign tumor, it can result in amenorrhea, galactorrhea, loss of libido, subfertility, osteoporosis, visual impairment, etc. due to the overproduction of prolactin (PRL) and the direct tumor mass effect. It has serious effects on the quality of life of the patients.

Currently, dopamine agonists (DAs), including bromocriptine (BRC) and cabergoline (CAB), are the first-line treatment for prolactinoma worldwide. Although DAs are effective in most patients with prolactinoma, the overall prevalence of drug resistance can be as high as 20–30% for BRC and 10% for CAB [4]. In addition to drug resistance, some people discontinue DAs because of intolerance to various severe side effects [5]. Some specific clinical circumstances, such as sudden or progressive visual loss and spontaneous or DA-induced cerebrospinal fluid (CSF) rhinorrhea, are also indications for surgery [6].

Hypertension is a prevalent cardiovascular disease and the most frequent modifiable risk factor associated with cardiovascular morbidity and mortality [7]. Studies have suggested that tumors secreting growth hormone (GH) and adrenocorticotropic hormone (ACTH) can cause hypertension [2,8]. However, this relationship remains unclear in other subtypes of PitNETs such as prolactinoma. The epidemiology of hypertension in patients with prolactinoma remains unclear. Nevertheless, previous studies have suggested that prolactin (PRL) can significantly increase blood pressure (BP) in rats [9]. However, the direct relationship between BP and prolactinoma is unclear, and whether BP can be altered by surgical and drug treatments remains unknown. In the current study, we report alterations in BP after corresponding treatments in patients and rodents with prolactinoma.

## 2. Materials and Methods

### 2.1. Study Design and Participants

This retrospective study reviewed patients who underwent surgery via the transsphenoidal approach for prolactinoma in our department between January 2005 and December 2022. This study was approved by the Ethical Review Board of Ruijin Hospital, which is affiliated with the Shanghai Jiao Tong University School of Medicine (PRLomaBP, 20240102125623800). All the surgeries were performed by two experienced neurosurgeons (Prof. W.Z. and Prof. Z.B.W.). The diagnosis of prolactinoma was based on clinical presentation, hyperprolactinemia, clear sellar regional tumor imaging on enhanced pituitary magnetic resonance imaging (MRI), and postoperative histopathological results. Surgical treatment was offered after the failure or intolerance of medical treatment, acute visual loss, and noninvasive prolactinomas with well-defined margin masses on MRI-imaging modalities [10,11]. Neuroendocrine hormone levels were assessed before and after surgery in all patients. Medical records were reviewed, and several data, including age, sex, diagnosis of hypertension, disease duration, tumor size, invasion, sensitivity to DAs, recurrence, and PRL levels were recorded.

### 2.2. BP Measurement in Humans

Blood pressure was measured 1 day before and 5 days after the transsphenoidal surgery. On the day of measurement, the BP was manually checked three times at 6:00 a.m., 2:00 p.m., and 6:00 p.m. The mean of these three measurements was recorded as the BP value for that day. The systolic and diastolic pressures were recorded separately.

### 2.3. Animals

Female F344 rats and nude mice were purchased from the Experimental Animal Center of the Chinese Academy of Sciences (Shanghai, China). All animal experimental procedures were approved by the Ethical Review Board of Ruijin Hospital, affiliated with the Shanghai Jiao Tong University School of Medicine, and were performed in strict accordance with the US National Institutes of Health Guidelines. The rodents were housed under specific pathogen-free conditions, including sterilized food and water. All efforts were made to reduce the total number of rodents used and to minimize their suffering.

### 2.4. In Situ Rat Prolactinoma Model

A total of 24 female rats were randomly divided into three groups: (i) sham (*n* = 8), (ii) control (Ctrl) (prolactinoma, *n* = 8), and (iii) CAB (prolactinoma + CAB, *n* = 8). To establish the in situ rat prolactinoma model, 1 cm silastic capsules containing 10 mg of 17-β estradiol (MedChemExpress, Shanghai, China, cat. E2758) were implanted into 4-week-old rats subcutaneously, as described in our previous reports [12,13]. Five weeks later, an MRI was performed to validate the establishment of prolactinoma. At this point, the CAB (0.5 mg/kg, MedChemExpress, Shanghai, China) or control (Ctrl) 0.9%, saline (100 μL) was injected twice a week by gavage. Four weeks later, an MRI examination was conducted to measure tumor size, and the BP was subsequently measured.

### 2.5. Xenograft Mouse Model

Eighteen female nude mice were randomly divided into three groups: (i) sham (*n* = 6), (ii) control (prolactinoma, *n* = 6), and (iii) CAB (prolactinoma + CAB, *n* = 6). To establish the xenograft models, MMQ cells (2 × 10^6^, ATCC CRL-10609, American Type Culture Collection, Manassas, VA, USA) were injected subcutaneously (up to a volume of 100 μL) in the flank. CAB was administered at a dosage of 0.75 mg/kg daily through gavage administration. After 14 days, all mice were euthanized, and the corresponding data were analyzed.

### 2.6. Rodent BP Measurement

Rat and mouse BP measurements were performed using a noninvasive Medlab system for rodents (Kew Technologies, Nanjing, China). Awake animals were allowed to acclimatize to the experimental conditions for approximately 2 h prior to the measurements. The BP was determined by averaging at least five consecutive measurements in a 30 min session.

### 2.7. Enzyme-Linked Immunosorbent Assay (ELISA)

Blood samples were harvested from the orbital venous plexus of rats and left hearts of mice prior to sacrifice and were further centrifuged at 2000 rpm for 20 min at 4 °C. Serum protein levels of PRL in different rodent groups were measured using the rat (cat. D731023; Sangon Biotech Co., Ltd., Shanghai, China) and mice (cat. DY1445; R&D Systems, Minneapolis, MN, USA) PRL ELISA kits according to manufacturer’s instructions. A spectrophotometer was used to detect absorbance at 450 nm.

### 2.8. Statistical Analysis

SPSS 25.0 (SPSS Inc., Chicago, IL, USA) and Graphpad Prism 8.0 (GraphPad Software, San Diego, CA, USA) were used to analyze the results. Comparisons between different groups were performed by the chi-square test and Student’s *t*-test for categorical variables. Correlations were assessed using Pearson’s correlation coefficient. All quantitative data were expressed as mean ± standard deviation (SD). *p* < 0.05 was considered to indicate a statistically significant difference.

## 3. Results

### 3.1. Baseline Characteristics

A total of 211 patients with prolactinoma underwent surgery during the study period, and 162 patients were identified. The remaining 49 patients were excluded due to incomplete data or adjustments in antihypertensive medication. The baseline characteristics of the patients are summarized in Table 1. Of the 162 patients, 37 were male and 125 were female (13–76; mean, 38.5 ± 13.2 years old). There were 85 microadenomas, 69 macroadenomas, and 8 giant adenomas. The mean disease duration was 26.7 ± 33.0 months. A total of 58 patients had a PRL value > 200 μg/mL. Thirty-nine patients had a history of hypertension. There were no changes in antihypertensive medication use during the pre- and postoperative periods. As shown in Table 1, age, sex, tumor size, invasion, and sensitivity to DAs were significantly associated with hypertension in patients with prolactinoma (all *p* < 0.05).

### 3.2. Alterations in BP before and after Surgery in Patients with Prolactinoma

**Figure 1:**
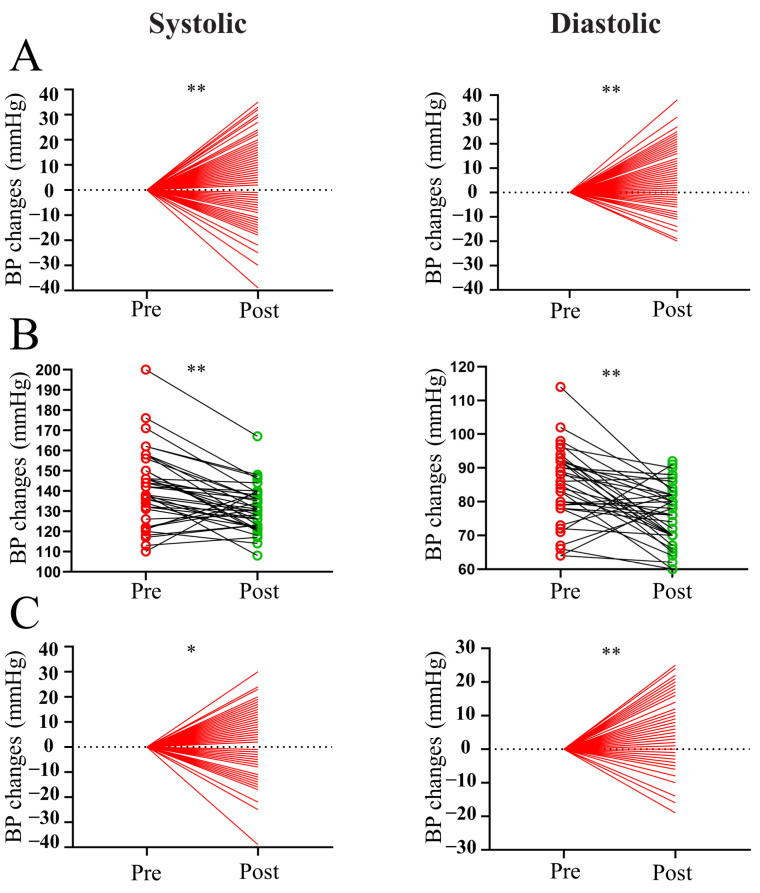
Pre- and postoperative BP changes in patients with prolactinoma. (**A**) Pre- and postoperative measurements of systolic and diastolic BP in all patients with prolactinoma. (**B**) Pre- and postoperative measurements of systolic and diastolic BP in hypertensive patients with prolactinoma. (**C**) Pre- and postoperative measurements of systolic and diastolic BP in nonhypertensive patients with prolactinoma. ** *p* < 0.01 vs. preoperative group; * *p* < 0.05 vs. preoperative group. Red indicates pre-operation and green indicates post-operation.

To observe the effect of surgery on BP in patients with prolactinoma, we analyzed pre- and postoperative BP changes in patients with or without hypertension. As shown in Figure 1A, the surgery induced a significant decrease in both systolic and diastolic BP (both *p* < 0.01). Furthermore, both systolic and diastolic BP were significantly reduced in the subdivided hypertensive (Figure 1B, both *p* < 0.01) and nonhypertensive groups (Figure 1C, both *p* < 0.05).

### 3.3. Alterations in BP before and after Surgery in Patients with or without Hormone Control

**Figure 2:**
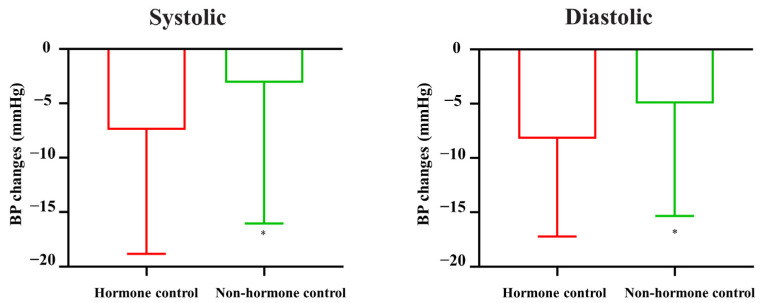
BP changes in patients with or without hormone control. * *p* < 0.05 vs. hormone control group.

Given that hypertension may be primarily caused by highly secreted hormones in PitNETs and not by tumor volume, we focused mainly on the hormone control group. As shown in Table 2, BPs significantly decreased in both the hormone control and nonhormone control groups (all *p* < 0.01). Moreover, there was a significantly higher decrease in systolic and diastolic BP in the hormone control group than in the nonhormone control group (Figure 2, both *p* < 0.05).

### 3.4. Variables Associated with BP Alternations before and after Surgery in Patients

To assess the effect of surgery on pre- and postoperative changes in BP in patients with prolactinoma, we analyzed the potentially related variables. As shown in Table 3, there was a significant reduction in diastolic BP in all youth (18–44), middle-aged (45–59), and elderly (>60 years) groups following surgery (all *p* < 0.05). Moreover, a significant decrease in systolic BP was observed in the youth group (*p* < 0.01). Furthermore, surgery markedly decreased both systolic and diastolic BP in patients with prolactinoma, with the following characteristics: female sex, macroadenoma, noninvasion, nonrecurrence, and preoperative PRL < 200 ng/mL (Table 3, all *p* < 0.05). In addition, surgery significantly decreased diastolic BP in those with the following characteristics: male sex, microadenoma, invasion, sensitivity to DAs, and preoperative PRL > 200 ng/mL (Table 3, all *p* < 0.05).

### 3.5. Variables Associated with BP Alternations before and after Surgery in Patients with Hypertension

Because prolactinoma may be highly associated with hypertension, we further analyzed these variables in patients with hypertension. As shown in Table 4, significant systolic and diastolic changes in BP after surgery were linked to several variables, including age, male sex, macroadenoma, invasion, and preoperative PRL < 200 ng/mL (all *p* < 0.05). In addition, significant diastolic BP changes following surgery were linked to some variables, including age, giant adenoma, noninvasion, and preoperative PRL > 200 ng/mL (all *p* < 0.05).

### 3.6. Alterations in BP Measurements before and after CAB Treatment in Rodent Prolactinoma Models

**Figure 3:**
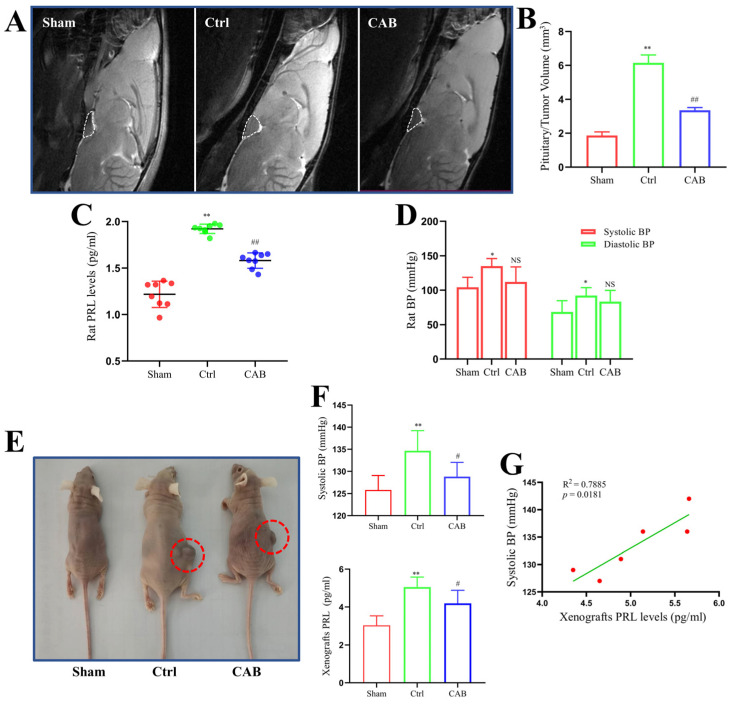
BP changes in rodent prolactinoma models. (**A**) Representative MRI images of in situ prolactinoma in each group. (**B**) Pituitary/tumor volumes of in situ prolactinomas in different rat groups; *n* = 4. (**C**) Serum PRL levels in different in situ prolactinoma groups; *n* = 8. (**D**) Systolic and diastolic BP in different in situ prolactinoma groups; *n* = 4. (**E**) Representative photos of xenograft prolactinoma in each group. The red circles shows the xenograft tumors. (**F**) Systolic BP and serum PRL levels in different xenograft prolactinoma groups; *n* = 6. (**G**) Correlation between changes in systolic BP and serum PRL level in the xenograft prolactinoma (Ctrl) group; *n* = 6. The values are presented as mean ± SD; * *p* < 0.05 vs. sham group; ** *p* < 0.01 vs. sham group; # *p* < 0.05 vs. Ctrl group; ## *p* < 0.01 vs. Ctrl group; NS, no significance.

To investigate the effects of prolactinoma and corresponding drug treatment on BP, we used in situ rat prolactinoma and xenograft mouse models, respectively. As shown in Figure 3A, we established a rat prolactinoma in situ model by injecting estrogen. MRI examination indicated that the volumes of tumors in rats treated with CAB were significantly lower than those in control rats (Figure 3B, *p* < 0.01). As expected, CAB significantly attenuated prolactinoma-induced upregulation of serum PRL (Figure 3C, *p* < 0.01). Notably, in situ, prolactinoma significantly increased both systolic and diastolic BP, whereas the elevated effects were slightly alleviated by CAB in rats without a statistical difference (Figure 3D, both *p* > 0.05). Moreover, no significant correlations were observed among systolic and diastolic BP and serum PRL levels in different groups.

We further used MMQ xenografts to mimic prolactinoma in vivo (Figure 3E). As shown in Figure 3F, there was a significant upregulation of systolic (not diastolic) BP and serum PRL levels on day 18 after the subcutaneous injection of MMQ cells (both *p* < 0.01). However, the effects of xenograft prolactinoma-induced increase in systolic BP and serum PRL levels were attenuated by CAB administration (Figure 3F, both *p* < 0.05). Furthermore, there was a significant positive correlation between systolic BP and serum PRL levels in the control group (Figure 3G, *p* < 0.05).

## 4. Discussion

Through clinical and preclinical studies, this study revealed that surgical or drug therapy could effectively decrease BP in patients with prolactinoma. Specifically, our data suggested that (i) surgery significantly reduced both the systolic and diastolic BP in patients with prolactinoma; (ii) both systolic and diastolic BP were significantly reduced in patients with prolactinoma with or without PRL hormone control following surgery; (iii) those with hormone control had a more significant reduction in both the systolic and diastolic BP when compared to patients without hormone control following surgery; (iv) operation-induced BP decline was significantly associated with several characteristics, including age, sex, tumor size, invasion, DAs-sensitivity, and preoperative PRL level; and (v) rodent prolactinoma models induced significant BP elevations, which were alleviated by CAB treatment.

Several subtypes of PitNETs can result in systemic complications, including hypertension, diabetes mellitus, obesity, and some other morbidities. Studies have suggested that any morbidity, such as hypertension, caused by the abnormal secretion of hormones needs to be treated appropriately [2,8]. To date, there are no studies on the association between prolactinoma and hypertension, although several studies have demonstrated the association between hyperprolactinemia and hypertension. Animal studies have shown that high PRL levels exert positive chronotropic and vasoconstrictive effects, which induce hypertension [9]. Subsequent mechanisms have been proposed to explain the hypertensive effects of PRL, including smooth-muscle cell proliferation [14], nitric oxide synthase modulation [15,16], low-grade inflammation, and inflammatory cell adhesion to the endothelium [17,18]. Recently, hyperprolactinemia was shown to be associated with hypertension in humans. Epidemiological data revealed that PRL levels > 8.0 ng/mL had 100% sensitivity for predicting high-peripheral BP in postmenopausal women [19,20]. Moreover, diurnal fluctuations in PRL levels were significantly associated with hypertension in men [21]. In contrast, PRL is associated with the incidence of cardiovascular events [22,23]. Although these results suggest that high PRL levels are closely associated with hypertension, there is no direct data to show that prolactinoma is linked to hypertension. In this study, we provide direct evidence that prolactinoma induced a significant mean systolic BP elevation of 30.71 mmHg in rats and 8.00 mmHg in mice. The cause-and-effect association can be elucidated by the significant elevation of PRL levels of 1.80 and 1.67 times secreted by in situ and xenograft tumors in rats and mice, respectively. Notably, a previous study suggested that a three-fold increase in plasma PRL can significantly increase BP in male mice with a single-copy transgene, which enables the inducible hepatic production of PRL and its cleavage product [24]. Although the PRL did not increase by three-fold in our study, it was sufficient to significantly increase BP. This phenomenon can be explained by the use of different rodent models; we employed in situ and xenograft female-rat and nude-mice prolactinoma models. Together, these data suggest that prolactinoma may result in an increase in BP via the oversecretion of PRL.

Given the role of PRL in BP increase, we further analyzed the BP changes following corresponding treatments, including surgery and DAs for prolactinoma. Regarding surgery, both systolic and diastolic BP in all patients showed a significant reduction when serum PRL returned to normal levels following surgery. This may be because each 5 mg/dL increase in PRL, even at normal levels, is associated with a significant increase in hypertension [23]. Thus, there was a mean decrease of 3.12 and 5.00 mmHg, respectively, in systolic and diastolic BP in the nonhormone control group, even though the hormone did not return to normal levels. The surgery-induced decline in PRL hormone levels was sufficient to significantly reduce BP. Moreover, the reductions in both systolic and diastolic BP were more obvious in the hormone control groups than in the nonhormone control group. This phenomenon may be due to a greater reduction and normalization of PRL in the hormone control group because of the success of the surgery.

More recently, another study [25] and our publications [10,11] have suggested that surgery can be considered a viable alternative first-line treatment for some patients with prolactinoma, especially noninvasive microadenomas. Prior data have shown that hormone remission rates following surgery are closely associated with tumor size, preoperative PRL levels, invasion, and resistance to DAs [26,27,28,29]. Our data consistently suggest that BP reduction is closely associated with these characteristics, which directly affect the operative outcomes of prolactinoma and hormone remission. Regarding the multiplied operative difficulty for total resection in recurrent prolactinoma, it is reasonable that BP showed no significant difference between pre- and postoperative values. Additionally, we found that surgery induced a significant decrease in BP in the youth (age 18–44), who account for the majority of patients with prolactinoma. Interestingly, we found a significant decrease in BP in females than in males. Yoo et al. reported that male patients with prolactinoma have larger tumors with more aggressive features. Males are less likely to undergo gross total resection and PRL normalization following surgery than females [30]. Additionally, a significant decrease in systolic BP was observed in patients with and without invasion. In addition, no statistical significance was observed in changes in diastolic BP, regardless of sensitivity to DA. Together, these data indicate that surgery could significantly decrease both systolic and diastolic BP, which were highly associated with the subdivided variables of age, sex, tumor size, invasion, resistance to DA, recurrence, and preoperative PRL levels.

Based on the baseline characteristic data of the 162 patients with prolactinoma, we found that sensitivity to DA was significantly associated with hypertension. Although adverse events of orthostatic hypotension have been reported in DAs, their role in BP, especially in hypertension, remains to be established. In a double-blind placebo-controlled crossover study, BRC was reported to be ineffective in lowering BP in nonprolactinoma patients with hypertension [31]. In another clinical study, BRC significantly reduced both lying and standing BP when combined with methyldopa in patients with hypertension [32] and reduced BP in hypertensive patients with Parkinson’s disease [33]. These data indicate that the ability of BRC to decrease BP depends on certain conditions. More recently, Kabootari et al. [34] demonstrated that another, more mainstream, DA for prolactinoma, CAB, showed no change in BP, except for a decrease in diastolic BP only among female patients after a 6-month follow-up. However, low BP is a classical side effect of CAB. In addition, Humphrey et al. [35] found that postpartum women who received 1 mg CAB had a lower mean systolic and diastolic BP at all time intervals. In this study, we investigated whether CAB reduced BP in prolactinoma using preclinical in situ rat and xenograft nude-mice prolactinoma models. In rats, CAB demonstrated a slightly decreased efficacy in in situ prolactinoma-induced BP upregulation, although the difference was not statistically significant. In xenograft mice, CAB significantly decreased BP, which positively correlated with serum PRL levels. The difference in results may be due to the differences in the subjects studied.

Our previous study suggested that the multidisciplinary team (MDT) approach has been employed in an attempt to bring about collaborative decision-making and concentrate clinical experience from multiple specialties on single-patient cases in a systematic fashion [36]. Thus, the MDT, including a neurosurgeon as the team leader in combination with experts from the departments of Endocrinology, Cardiovascular medicine, Hypertension, Pharmacy, and Anesthesiology, is recommended to discuss the BP management strategies after the initial clinical diagnosis in patients with prolactinoma, especially for those with abnormal BP.

The limitations of this study include retrospective biases, single-center study, and the relatively small number of patients in the group analysis. In addition, we did not analyze the effects of CAB on BP in patients with prolactinoma. Moreover, a 24 h blood-pressure method in a long-term after operation should be employed. Future prospective studies with larger sample sizes from multiple centers are required to confirm our findings.

## 5. Conclusions

In patients with prolactinoma, surgery can decrease BP in patients with or without hypertension. This BP-lowering effect was more prominent with age, sex, tumor size, invasion, resistance to DAs, recurrence, and preoperative PRL levels. In addition, our preclinical studies suggested that prolactinoma could induce significant BP elevation, which was attenuated by CAB treatment. Furthermore, an MDT is recommended to facilitate preoperative and postoperative management to avoid abnormal BP-induced adverse events in patients with prolactinoma.

## Figures and Tables

**Table 1:** Baseline characteristics in 162 patients with prolactinoma.

Baseline Characteristics				
Hypertension				*p* Value
	Total (*n* = 162)	Y (*n* = 39)	N (*n* = 123)	
Age				
	38.45 ± 13.29	51.08 ± 11.77	34.45 ± 10.98	0.0001
Gender				
Female	125(77.16%)	22 (56.41%)	103 (83.74%)	0.0003
Male	37 (22.84%)	17 (43.59%)	20 (16.26%)	
Duration				
	36.59 ± 56.52	25.87 ± 73.50	32.52 ± 32.44	0.1451
Size				
Microadenoma	57 (35.19%)	8 (20.51%)	49 (39.84%)	0.0197
Macroadenoma	92 (56.79%)	26 (66.67%)	66 (53.66%)	
Giant adenoma	13 (8.02%)	5 (12.82%)	8 (6.50%)	
Invasion				
Yes	48 (29.63%)	18 (46.15%)	30 (24.39%)	0.0001
No	114 (70.37%)	21 (53.85%)	93 (75.61%)	
DAs resistance				
N	31 (19.14%)	4 (10.26%)	27 (21.95%)	0.0017
Y	66 (40.74%)	12 (30.77%)	54 (43.90%)	
N/A	65 (40.12%)	23 (58.97%)	42 (34.15%)	
Recurrence				
Y	4 (2.47%)	2 (5.13%)	2 (1.63%)	0.2219
N	158 (97.53%)	37 (94.87%)	121 (98.37%)	
Preoperative PRL level				
>200 ng/mL	58 (35.80%)	16 (41.03%)	42 (34.15%)	0.4380
<200 ng/mL	104 (64.20%)	23 (58.97%)	81 (65.85%)	

**Table 2:** BP before and after surgery in 162 patients with or without hormone control.

BP Measurements Before and after Operation in Patients with or without Hormone Control				
All Patients	Preoperative, mmHg	Postoperative, mmHg	Difference (Post–Pre), mmHg	*p* Value
Systolic pressure	116.96 ± 19.06	111.68 ± 15.82	−5.28 ± 12.34	0.000
Diastolic pressure	73.75 ± 11.79	67.13 ± 9.97	−6.62 ± 9.79	0.000
Hormone control (*n* = 75)				
Systolic pressure	118.05 ± 19.28	110.60 ± 15.43	−7.44 ± 11.40	0.000
Diastolic pressure	74.21 ± 12.11	65.96 ± 9.37	−8.25 ± 8.98	0.000
Nonhormone control (*n* = 67)				
Systolic pressure	115.88 ± 18.90	112.75 ± 16.22	−3.12 ± 12.93	0.000
Diastolic pressure	73.30 ± 11.53	68.30 ± 10.47	−5.00 ± 10.34	0.000

**Table 3:** BP changes before and after surgery in 162 patients with different variables.

	Preoperative, mmHg	Postoperative, mmHg	Difference (Pre–Post), mmHg	*p* Value
Age				
<18 (*n* = 6)	108.33 ± 11.48	106.17 ± 14.72	−2.17 ± 11.72	0.7820
	67.00 ± 14.42	61.00 ± 14.14	−6.00 ± 12.70	0.4835
18–44 (*n* = 111)	112.41 ± 15.33	108.16 ± 13.47	−4.24 ± 12.21	0.0295
	71.73 ± 10.55	65.45 ± 8.95	−6.28 ± 9.11	0.0001
45–59 (*n* = 32)	124.28 ± 18.08	117.53 ± 16.04	−6.75 ± 10.68	0.1192
	77.66 ± 10.33	71.53 ± 10.79	−6.13 ± 10.18	0.0237
>60 (*n* = 13)	141.85 ± 27.91	129.85 ± 18.98	−12.00 ± 16.00	0.2121
	84.54 ± 16.02	73.46 ± 8.68	−11.08 ± 12.92	0.0383
Gender				
Male (*n* = 37)	125.38 ± 22.46	117.24 ± 17.61	−8.14 ± 14.68	0.0872
	80.22 ± 12.10	70.62 ± 10.68	−9.59 ± 10.68	0.0006
Female (*n* = 125)	114.47 ± 17.26	110.03 ± 14.93	−4.44 ± 11.49	0.0306
	71.84 ± 11.04	66.10 ± 9.56	−5.74 ± 9.38	0.0001
Size				
Microadenoma	113.91 ± 13.13	108.76 ± 14.17	−5.15 ± 10.26	0.0529
(*n* = 57)	72.33 ± 8.27	66.27 ± 9.91	−6.05 ± 9.02	0.0008
Macroadenoma	119.07 ± 21.61	113.23 ± 16.01	−5.84 ± 13.42	0.0398
(*n* = 92)	74.74 ± 13.47	67.36 ± 10.10	−7.38 ± 10.03	0.0001
Giant adenoma	119.00 ± 19.13	117.57 ± 15.28	−1.43 ± 11.15	0.8350
(*n* = 13)	76.50 ± 9.91	71.71 ± 8.61	−4.79 ± 10.36	0.2005
Invasion				
Yes (*n* = 48)	122.65 ± 19.93	117.56 ± 14.79	−5.08 ± 13.78	0.1635
	76.48 ± 10.80	70.52 ± 8.77	−5.96 ± 10.44	0.0042
No (*n* = 114)	114.57 ± 18.07	109.20 ± 15.51	−5.37 ± 11.62	0.0174
	72.61 ± 11.95	65.70 ± 10.06	−6.90 ± 9.45	0.0001
DAs-resistance				
Yes (*n* = 66)	114.47 ± 15.98	109.73 ± 15.01	−4.74 ± 12.50	0.0835
	73.17 ± 11.00	66.59 ± 10.78	−6.58 ± 8.97	0.0008
No (*n* = 31)	112.29 ± 13.00	106.68 ± 13.27	−5.61 ± 9.61	0.1031
	71.45 ± 8.97	66.19 ± 8.76	−5.26 ± 9.37	0.0251
N/A (*n* = 65)	121.72 ± 22.82	116.05 ± 16.50	−5.68 ± 13.19	0.1093
	75.45 ± 13.33	68.12 ± 9.49	−7.32 ± 10.60	0.0005
Recurrence				
Yes (*n* = 4)	127.00 ± 21.75	116.25 ± 13.08	−10.75 ± 9.83	0.4908
	80.25 ± 10.50	66.00 ± 2.45	−14.25 ± 8.84	0.0620
No (*n* = 158)	116.71 ± 18.86	111.56 ± 15.81	−5.15 ± 12.33	0.0092
	73.59 ± 11.74	67.16 ± 10.06	−6.43 ± 9.70	0.0001
Serum PRL				
>200 ng/mL	118.47 ± 20.59	113.14 ± 15.71	−5.33 ± 13.41	0.1232
(*n* = 58)	73.66 ± 12.26	68.26 ± 10.00	−5.40 ± 10.00	0.0113
<200 ng/mL	116.13 ± 18.01	110.87 ± 15.74	−5.26 ± 11.64	0.0267
(*n* = 104)	73.81 ± 11.47	66.50 ± 9.86	−7.31 ± 9.56	0.001

**Table 4:** Change in BP before and after surgery in 39 hypertensive patients with different variables.

Variable	Preoperative, mmHg	Postoperative, mmHg	Difference (Post–Pre), mmHg	*p* Value
Age				
18–44 (*n* = 14)	137.07 ± 15.34	129.00 ± 6.66	8.07 ± 17.00	0.0825
	83.93 ± 15.14	76.50 ± 10.68	7.43 ± 13.69	0.1456
45–59 (*n* = 15)	139.80 ± 13.80	129.87 ± 9.53	9.93 ± 9.89	0.0295
	84.60 ± 9.95	76.00 ± 9.33	8.6 ± 10.68	0.0212
>60 (*n* = 10)	150.40 ± 25.95	135.80 ± 16.67	14.60 ± 16.16	0.1517
	88.40 ± 16.14	75.50 ± 8.21	12.90 ± 12.95	0.0370
Gender				
Male (*n* = 17)	143.94 ± 18.14	130.35 ± 10.73	13.59 ± 16.03	0.0147
	88.53 ± 10.54	76.47 ± 7.80	12.06 ± 11.05	0.0009
Female (*n* = 22)	139.68 ± 18.00	131.64 ± 11.04	8.05 ± 13.00	0.0880
	82.86 ± 14.53	75.73 ± 10.17	7.14 ± 13.30	0.0722
Size				
Microadenoma	133.00 ± 14.83	131.63 ± 8.76	1.38 ± 15.92	0.8358
(*n* = 8)	78.25 ± 12.48	77.13 ± 13.36	1.13 ± 12.06	0.8730
Macroadenoma	145.23 ± 18.32	130.54 ± 11.90	14.69 ± 12.54	0.0015
(*n* = 26)	87.88 ± 13.36	75.85 ± 8.15	12.04 ± 11.37	0.0003
Giant adenoma	136.00 ± 16.24	133.00 ± 8.12	3.00 ± 13.05	0.5414
(*n* = 5)	83.40 ± 8.50	75.40 ± 5.43	8.00 ± 13.68	0.0061
Invasion				
Yes (*n* = 18)	142.67 ± 17.18	130.06 ± 10.15	12.61 ± 13.93	0.0135
	84.89 ± 11.69	75.06 ± 6.87	9.83 ± 12.81	0.0052
No (*n* = 21)	140.57 ± 18.95	131.95 ± 11.47	8.62 ± 15.00	0.0895
	85.71 ± 14.43	76.90 ± 10.76	8.81 ± 12.41	0.0345
DAs-resistance				
Yes (*n* = 12)	139.00 ± 14.27	131.83 ± 7.69	7.17 ± 16.67	0.1567
	86.50 ± 10.32	79.00 ± 9.87	7.50 ± 11.00	0.0955
No (*n* = 4)	129.75 ± 11.23	124.50 ± 4.71	5.25 ± 12.87	0.1670
	75.00 ± 11.79	70.50 ± 9.10	4.50 ± 13.59	0.3271
N/A (*n* = 23)	144.91 ± 19.84	131.83 ± 12.61	13.09 ± 13.16	0.0123
	86.52 ± 14.03	75.48 ± 8.28	11.04 ± 12.86	0.0027
Serum PRL				
>200 ng/mL	142.31 ± 18.57	131.31 ± 10.67	11.00 ± 14.42	0.0558
(*n* = 16)	85.75 ± 11.26	77.50 ± 7.32	8.25 ± 11.84	0.0239
<200 ng/mL	141.00 ± 17.90	130.91 ± 11.09	10.09 ± 14.81	0.0297
(*n* = 23)	85.04 ± 14.46	75.04 ± 10.21	10.00 ± 13.07	0.0111

## Data Availability

The datasets in this study are available from the corresponding author upon reasonable request.
